# Global trends and neurobiological frontiers of manual therapy in sleep disorders: integrating bibliometrics with clinical evidence

**DOI:** 10.3389/fpsyt.2026.1863957

**Published:** 2026-06-24

**Authors:** Yixiang Wang, Donghai Wu, Yingjun Liu, Siying Qu, Yutian Chen, Huiqin Chu, Haiju Sun, Xiaoyu Li

**Affiliations:** 1 The Third Affiliated Hospital of Zhejiang Chinese Medical University (Zhongshan Hospital of Zhejiang Province), Hangzhou, Zhejiang, China; 2 Key Laboratory of Acupuncture and Neurology of Zhejiang Province, The Third Clinical Medical College, Zhejiang Chinese Medical University, Hangzhou, Zhejiang, China; 3 The First Affiliated Hospital of Zhejiang Chinese Medical University (Zhejiang Provincial Hospital of Traditional Chinese Medicine), Hangzhou, Zhejiang, China

**Keywords:** 5-HT, bibliometric analysis, HPA axis, manual therapy, sleep disorders

## Abstract

**Background:**

Sleep disorders not only impair nocturnal rest but also significantly compromise daytime functioning, emotional regulation, and overall mental well-being. Beyond conventional pharmacological treatments, manual therapy has emerged as a promising non-pharmacological intervention. Specifically, emerging evidence suggests its benefits may extend to alleviating psychological distress and enhancing mood. This study employs a bibliometric approach to systematically investigate the current status, research hotspots, and future trends of manual therapy for sleep disorders, with an emphasis on its psycho-physiological outcomes.

**Methods:**

Publications related to manual therapy for sleep disorders were retrieved from the Web of Science Core Collection (WoSCC). Bibliometric visualizations and analyses were conducted using VOSviewer and CiteSpace. Furthermore, clinical trial records from PubMed were extracted to assess the translational and clinical advancements in this field.

**Results:**

The analysis included 594 publications originating from 321 institutions across 63 countries. The overall trend demonstrates a consistent annual increase in both publication volume and citation impact, reflecting escalating academic interest. Keyword and literature co-occurrence analyses indicate that exploring neurobiological mechanisms and circadian rhythm regulation are the predominant research frontiers.

**Conclusion:**

Bibliometric evidence indicates that research on manual therapy for sleep disorders is evolving toward multidimensional and interdisciplinary integration. Manual therapy increasingly emerges as a key complementary treatment, exerting therapeutic effects via the regulation of 5-hydroxytryptamine (5-HT) and the Hypothalamic-Pituitary-Adrenal axis (HPA axis). Its safety and efficacy represent distinct advantages; however, future clinical translation necessitates multi-center validation and standardized sham-controlled protocols.

## 1 Introduction

Sleep is an essential physiological process that sustains physical health, neurocognitive performance, and mental well-being. In contrast, sleep disorders, which manifest as a range of functional disturbances across the sleep-wake cycle, are closely associated with emotional dysregulation and adverse mental health consequences. According to the International Classification of Sleep Disorders, Third Edition, Text Revision (ICSD-3-TR) released by the American Academy of Sleep Medicine (AASM) in 2023, sleep disorders are categorized into six major groups: insomnia, sleep-disordered breathing, central disorders of hypersomnolence, circadian rhythm sleep-wake disorders, parasomnias, and sleep-related movement disorders (1). Driven by profound shifts in living environments, modern social rhythms, and altered light exposure patterns, sleep disturbances have escalated into a global public health crisis. Patients with sleep disorders often demonstrate severe presence of somatizations and sensitization, which are closely intertwined with sleep disturbances (2). Typical somatic manifestations include dyspnea, tension headaches, palpitations, chest tightness, gastrointestinal dysfunction, and chronic musculoskeletal pain (3). Sensitization symptoms are mainly characterized by central sensitization, such as reduced pain threshold, exaggerated pain responses to mild stimuli, and abnormal pain induced by harmless stimuli (4). Somatization and sensitization not only aggravate the subjective suffering of sleep disorders but also severely damage physical and mental health, elevating the risks of cardiovascular instability, chronic pain syndromes, anxiety and depressive disorders, and neurodegenerative diseases (2, 5, 6). Epidemiological data indicate that chronic insomnia afflicts approximately 6% of the adult population in industrialized nations, with overall prevalence rates reaching 10% in Europe (2), 15% in China (7), and up to 15-24% in the United States (8). In addition to persistently high prevalence rates, poor sleep quality is also associated with complex bidirectional relationships involving sociodemographic factors, chronic physical illnesses, and mental disorders (9). Socioeconomic factors, health behaviors, comorbidities, and self-rated health are consistently linked to insomnia risk (10–12). Chronic sleep deprivation not only impairs immune and endocrine regulation but also significantly increases the risk of severe systemic morbidities, including cardiovascular events, stroke, asymptomatic cerebrovascular damage, and neurodegenerative conditions such as Alzheimer’s disease (AD) and related dementias (13).

Consequently, the global medical community is increasingly turning toward complementary and alternative medicine (CAM) to bridge these therapeutic gaps, particularly seeking interventions capable of simultaneously addressing the somatic and psychological dimensions of sleep disorders (14). Treatment options for sleep disorders include traditional medication, non-pharmacological approaches, and some less frequent ones, such as biofeedback, hypoglossal nerve stimulation, weighted blankets therapy and microbiome-targeted interventions (15–17). Manual therapy has emerged as a prominent non-pharmacological modality, utilizing precise physical techniques such as pressing, kneading, stretching, and joint manipulation (18). Crucially, contemporary research demonstrates that the benefits of manual therapy extend far beyond the mere modulation of sleep architecture. It exerts a profound holistic effect by alleviating psychological distress, reducing physiological hyperarousal, and directly enhancing mood. Operating within a biopsychosocial framework, meta-analyses have corroborated that manual therapy offers distinct advantages over conventional pharmacotherapy in improving long-term sleep outcomes for insomnia patients without the associated risk of chemical dependency (14). Despite its increasing clinical application, the psychological and neurobiological pathways through which manual therapy ameliorates sleep disturbances remain insufficiently mapped. While a few systematic reviews have evaluated its clinical efficacy, the global intellectual structure, evolutionary trajectories, and dynamic research hotspots in this field have yet to be comprehensively quantified. Although some bibliometric studies have preliminarily explored the application of manual therapy in the field of sleep medicine, this study differs from previous work in that his study utilizes prominent visualization tools (VOSviewer and CiteSpace) to systematically analyze the Web of Science Core Collection (WoSCC) database. Furthermore, by integrating clinical trial data from PubMed, this study aims to holistically visualize the global research trends, pinpoint core pathogenic mechanisms under investigation, and identify emerging frontiers, thereby providing a robust evidence-based reference for future translational research and clinical guideline development.

## 2 Methods

### 2.1 Data source and search strategy

**Figure 1:**
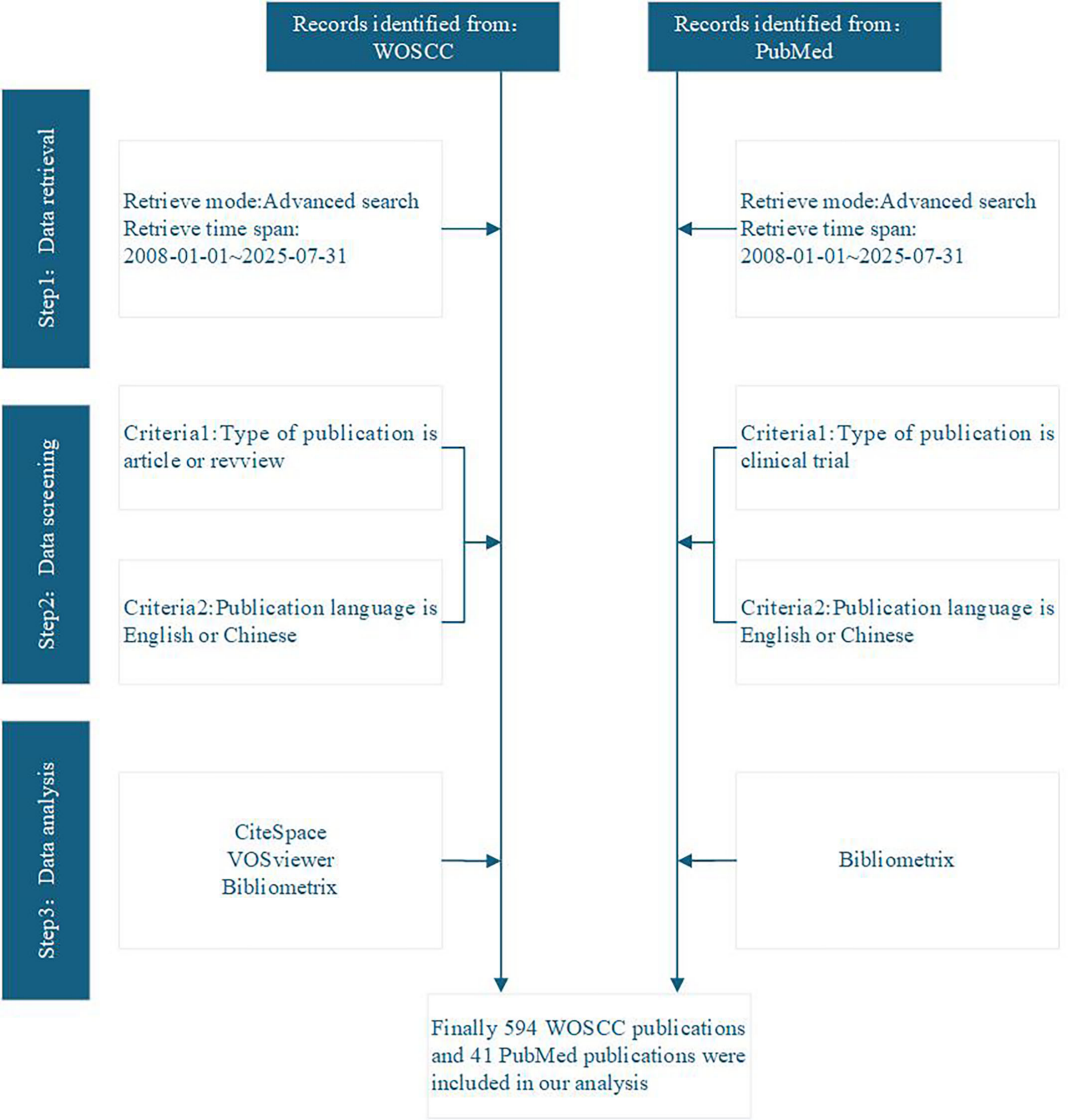
Literature search flowchart in research.

To ensure the high quality and standardized formatting of the retrieved literature, data were primarily sourced from the WoSCC and PubMed. WoSCC is internationally recognized as the optimal database for bibliometric studies due to its rigorous inclusion criteria and comprehensive citation indexing (19). Furthermore, clinical trial data from PubMed database were supplemented to assess clinical translation. To minimize selection bias, the search strategy combined the Topic (TS) field in WoSCC with a Title/Abstract search filtered for “Clinical Trials” in PubMed. The complete search strategies are detailed in **Table 1**. Strict inclusion and exclusion criteria were applied: (i) document types were strictly limited to original “Articles” and “Reviews”; (ii) publications were restricted to the English or Chinese language; and (iii) two independent researchers manually screened the titles and abstracts to eliminate false-positive results (e.g., studies where manual therapy or sleep disorders were merely mentioned in passing rather than being the core focus). Discrepancies were resolved through consensus. Following this rigorous screening, a final dataset of 594 valid WoSCC publications and 41 PubMed clinical trials was obtained for visualization. The detailed literature screening pipeline is illustrated in **Figure 1**.

**Table 1:** Search strategy in web of science core collection and PubMed.

Web of science core collection strategy	PubMed strategy
(TS=(Manual Therapy) OR TS=(Massage) OR TS=(Chiropractic) OR TS=(Mobilization) OR TS=(Spinal Manipulation) OR TS=(Myofascial Release) OR TS=(Tuina) OR TS=(Shiatsu)) AND (TS=(Sleep disorder) OR TS=(Somnipathy) OR TS=(Hypnosia) OR TS=(Sleep disturbance) OR TS=(insomnia) OR TS=(short duration sleep))	((((((((Manual Therapy[Title/Abstract]) OR (Massage[Title/Abstract])) OR (Chiropractic[Title/Abstract])) OR (Mobilization[Title/Abstract])) OR (Spinal Manipulation[Title/Abstract])) OR (Myofascial Release[Title/Abstract])) OR (Tuina[Title/Abstract])) OR (Shiatsu[Title/Abstract])) AND (((((((Sleep disorder[Title/Abstract])) OR (Somnipathy[Title/Abstract])) OR (Hypnosia[Title/Abstract])) OR (Sleep disturbance[Title/Abstract])) OR (insomnia[Title/Abstract])) OR (short duration sleep[Title/Abstract]))

### 2.2 Data analysis and visualization

Bibliometric mapping and quantitative analyses were collaboratively executed using three scientometric tools: VOSviewer (version 1.6.20), CiteSpace (version 6.4.R1), and the bibliometrix package in R software (version 4.5.1).

VOSviewer was utilized to construct complex co-occurrence networks based on a probabilistic modeling approach (20). Specifically, it was deployed to map spatial distribution and collaboration networks among highly productive countries, institutions, and core authors. Additionally, it performed keyword co-occurrence analysis, where nodes size reflects frequency and link strength denotes conceptual interrelatedness. CiteSpace, grounded in network dynamics, is uniquely advantageous for detecting temporal trends and intellectual turning points (21). It was configured for reference co-citation analyses and burst detection algorithms. The temporal slicing was set from 2008 to 2025, with a “Years Per Slice” of 1. The node type for co-citation network analysis was designated as “cited references”, and keyword burst detection was integrated to trace the evolution of research themes. Kleinberg’s citation burstness algorithm was applied to capture sudden spikes in citations, identifying emerging frontiers.

Additionally, the Bibliometrix R-tool provide a macroscopic statistical overview (22), evaluating annual scientific production dynamics and visualizing the evolutionary trends of thematic elements.

## 3 Result

### 3.1 Publication years

**Figure 2:**
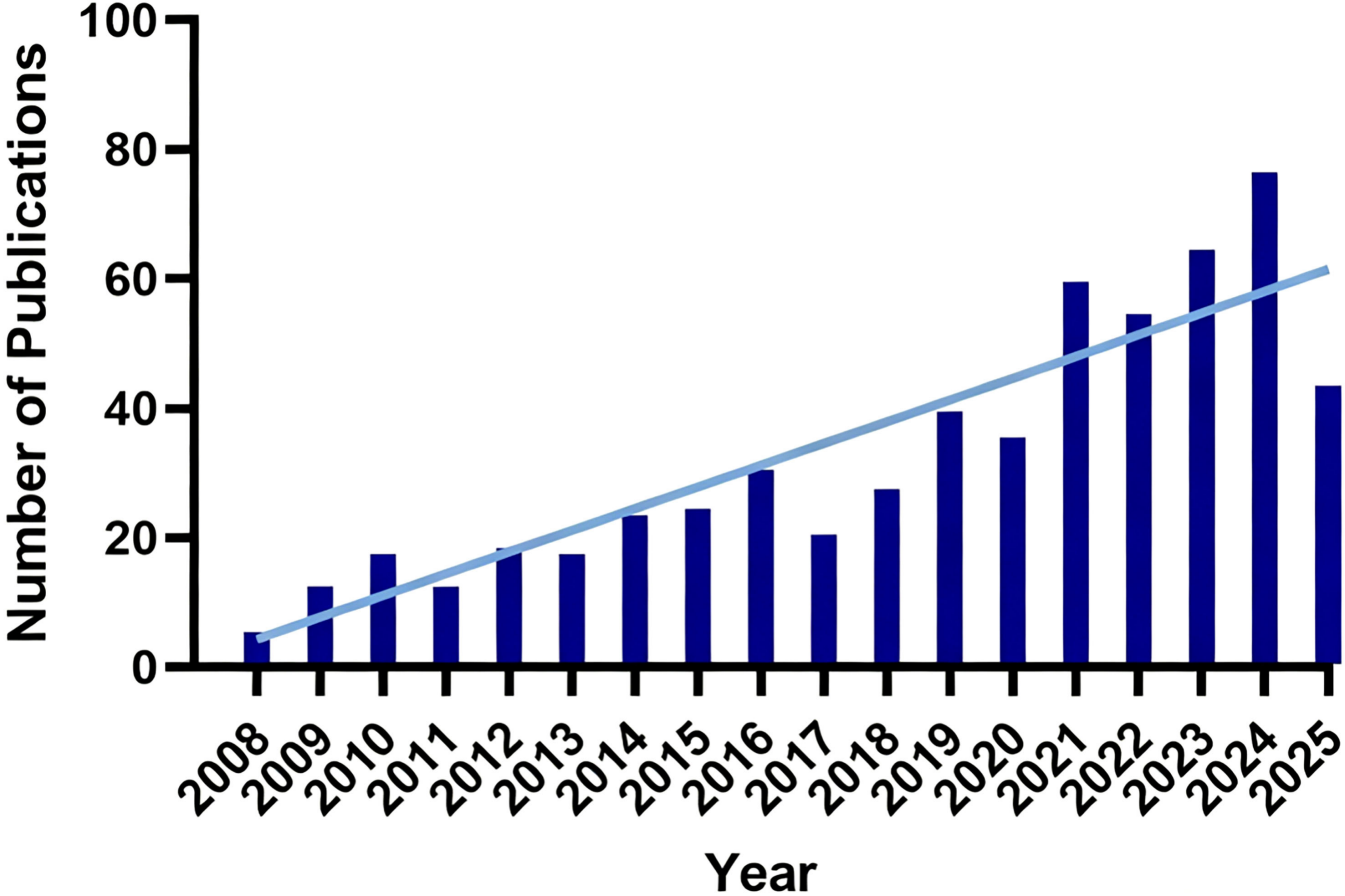
Publications of articles from 2008 to 2025.

Analyzing the evolution of publication timing is critical for understanding the developmental trajectory and focal shifts within a research field. This study identified 594 research papers published worldwide between January 1, 2008, and July 31, 2025, with their publication quantity trend clearly presented in **Figure 2**.

The temporal trend can be divided into three stages: Initial Exploration (2008-2010). Publication volume grew modestly from 6 papers in 2008 to 19 in 2010. Steady Growth (2011-2019). Following a brief dip in 2011, the research scale expanded steadily. Rapid Development (2020-2025). Although there was a slight fluctuation between 2017 and 2018, yet the overall trend remained a high-speed growth. The research peaked in 2024, with 83 papers published that year and total citations as high as 2748 times. The above-mentioned data indicate that manual therapy for sleep disorders has become a research hotspot that continues to attract attention in the current academic community. This also signals that the field will continue to evolve toward a deeper focus on mechanistic exploration and clinical translation.

### 3.2 Distribution of countries/regions and institutions

**Table 2** presents the top 10 most productive institutions. Harvard University ranks first with a significantly lead, establishing its core status in this field. By analyzing the institutional clusters and publication output rankings, we can gain clear insights into the distribution pattern of core research forces of manual therapy for sleep disorders.

**Table 2:** Top 10 institutions by number of publications.

Rank	Institution	Documents
1	Harvard University	12
2	Harvard University Medical Affiliates	9
3	Harvard Medical School	8
4	University of California System	8
5	University of Glasgow	8
6	Vrije Universiteit Amsterdam	8
7	Laval University	7
8	Mayo Clinic	7
9	Tsinghua University	7
10	Utrecht University	6

**Figure 3:**
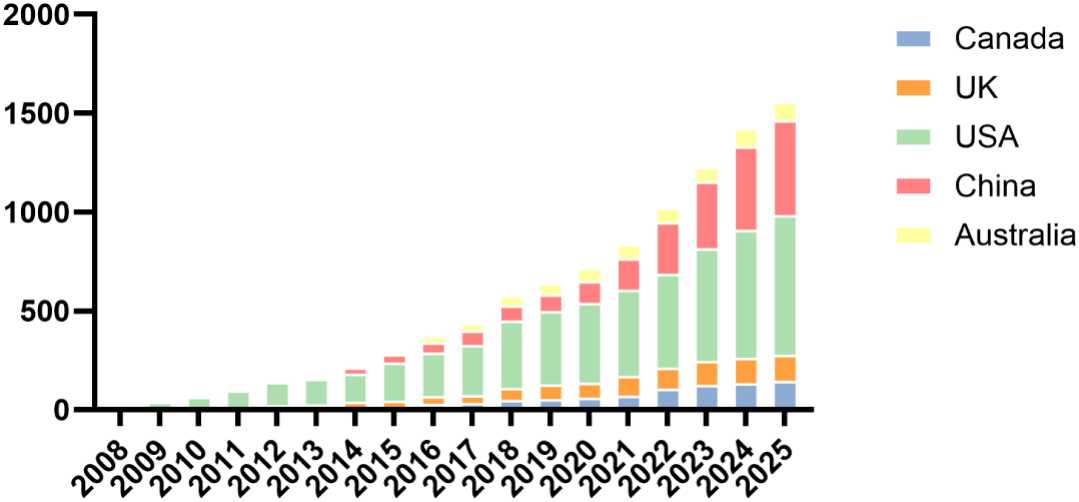
Top 5 national annual publishing trends from 2008 to 2025.

**Table 3** and **Supplementary Figure 1** illustrate the top 10 most productive countries. The United States and China are the two most productive countries. The United States and China fundamentally dominate global research output, contributing 152 and 108 publications, respectively. Together, they account for approximately 43.7% of the total global literature. **Figure 3** demonstrates that the number of annual publications in the top 5 most productive countries also shows a steady increase in the number of publications in each country related to manual therapy for sleep disorders. By analyzing the country-level research productivity and annual publication trends, we can gain clear insights into the global distribution of research forces and the dynamic development trend of manual therapy for sleep disorders worldwide.

**Table 3:** Top 10 countries or regions by number of publications.

Rank	Country or Region	Documents	Citations
1	USA	152	7722
2	China	108	1276
3	UK	54	3570
4	Canada	29	2026
5	Australia	28	3002
6	Italy	25	771
7	Brazil	24	477
8	Germany	23	1214
9	Spain	19	370
10	Iran	17	350

**Figure 4:**
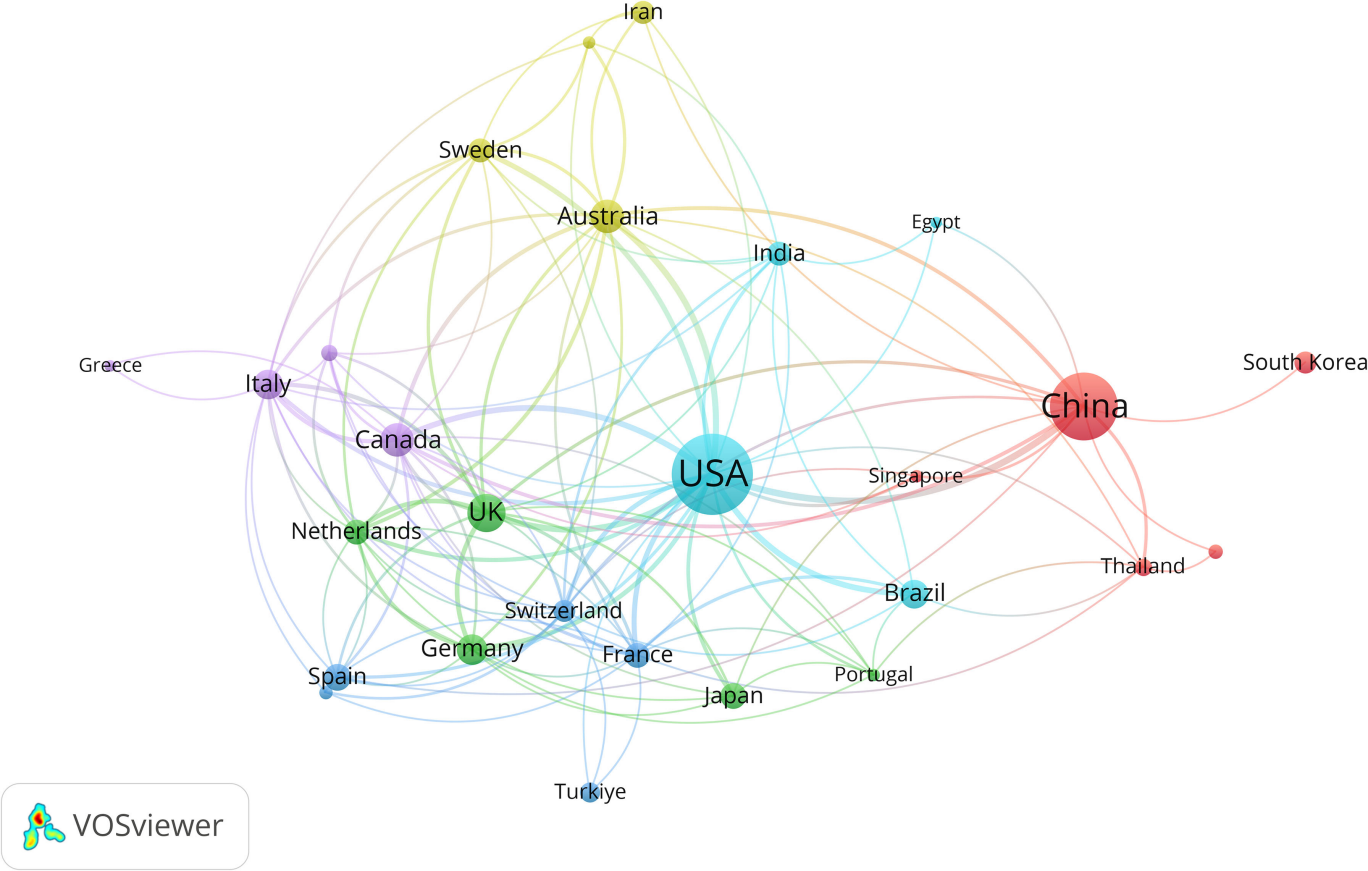
Network visualization of international relations.

To explore the relationship in international academic collaboration, VOSviewer was used to analyze collaboration relationships between countries. The resulting network graph is shown in **Figure 4**. This analysis only included countries and regions with no fewer than 5 publications, ultimately covering 27 research entities. Based on the ranking of Total Link Strength (TLS) obtained and analyzed by VOSviewer, the top five countries are USA (TLS = 107), UK (TLS = 51), Australia (TLS = 45), Canada (TLS = 41), and China (TLS = 36). These countries have critically led the formation and development of the international academic collaboration network in this field.

**Figure 5:**
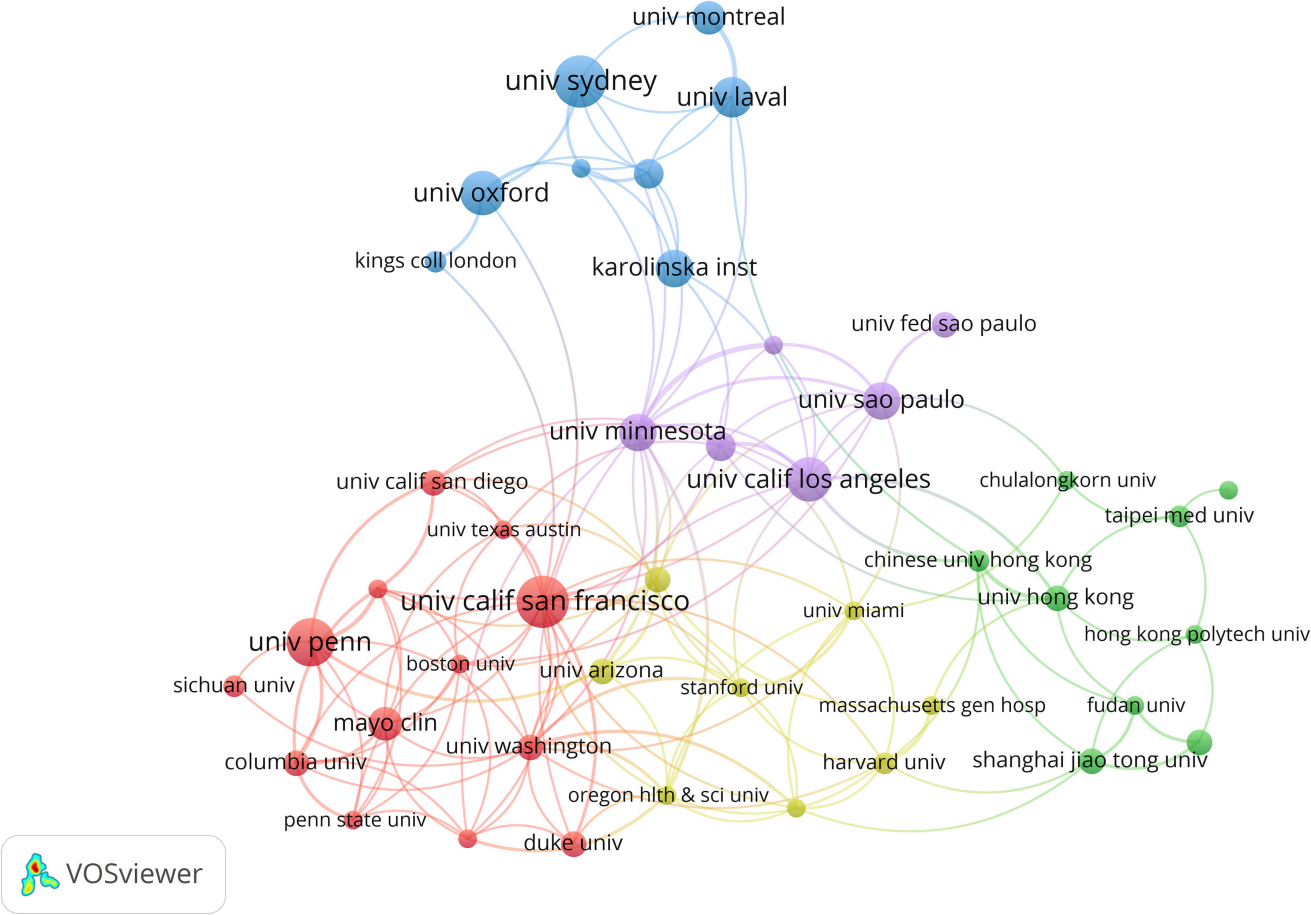
Web visualization of institutional citation analysis.

To academic connections at the institutional level reveals the core cooperative networks and influential forces within the field of manual therapy for sleep disorders, VOSviewer was similarly employed for citation relationship analysis, generating a collaborative network graph (**Figure 5**). The analysis was restricted to institutions with no fewer than 5 publications, encompassing a total of 44 eligible institutions. Ranked by Total Link Strength (TLS), the top three institutions are University of Minnesota (TLS = 22), University of California, San Francisco (TLS = 20), and University of Washington (TLS = 19). By examining the institutional collaboration network and the TLS rankings of these core institutions, we can gain clear insights into the distribution pattern of key research forces in manual therapy for sleep disorders.

### 3.3 Authors and co-citation authors

**Figure 6:**
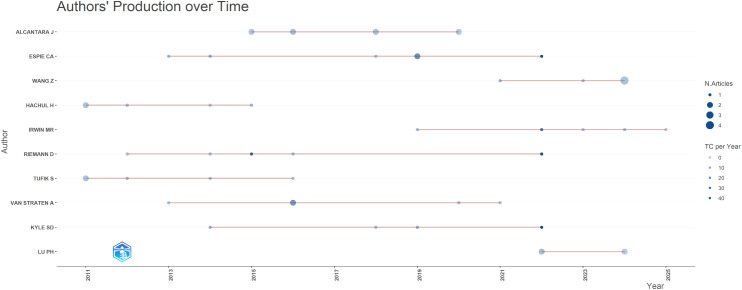
Top 10 authors’ production over time.

This study included a total of 594 levant literature items and involved 3,334 authors. Their research contributions collectively form the academic achievement system in the field of manual therapy for sleep disorders. **Figure 6** intuitively presents the distribution of literature output among authors in this field, while **Table 4** lists the 9 core authors with the highest research output. Among them, Espie Colin A. ranks first with 6 publications and 443 citations. Van Straten Annemieke and Irwin Michael R. follow closely, who both have published 5 papers with 193 and 137 citations, respectively. The research achievements of these authors highlight their outstanding contributions to the field. Their research directions and findings serve as a key reference for future in-depth exploration in this area, providing important guidance for subsequent researchers in identifying research priorities and establishing collaborative networks.

**Table 4:** Top 9 authors by number of publications.

Rank	Author	Documents	Citations	Total link strength
1	Espie Colin A.	6	443	9
2	Irwin Michael R.	5	137	4
3	Van Straten Annemieke	5	193	1
4	Alcantara Joel	4	34	6
5	Hachul Helena	4	82	3
6	Kyle Simon D.	4	295	6
7	Riemann Dieter	4	618	8
8	Schenck Carlos H.	4	21	0
9	Tufik Sergio	4	38	3

**Figure 7:**
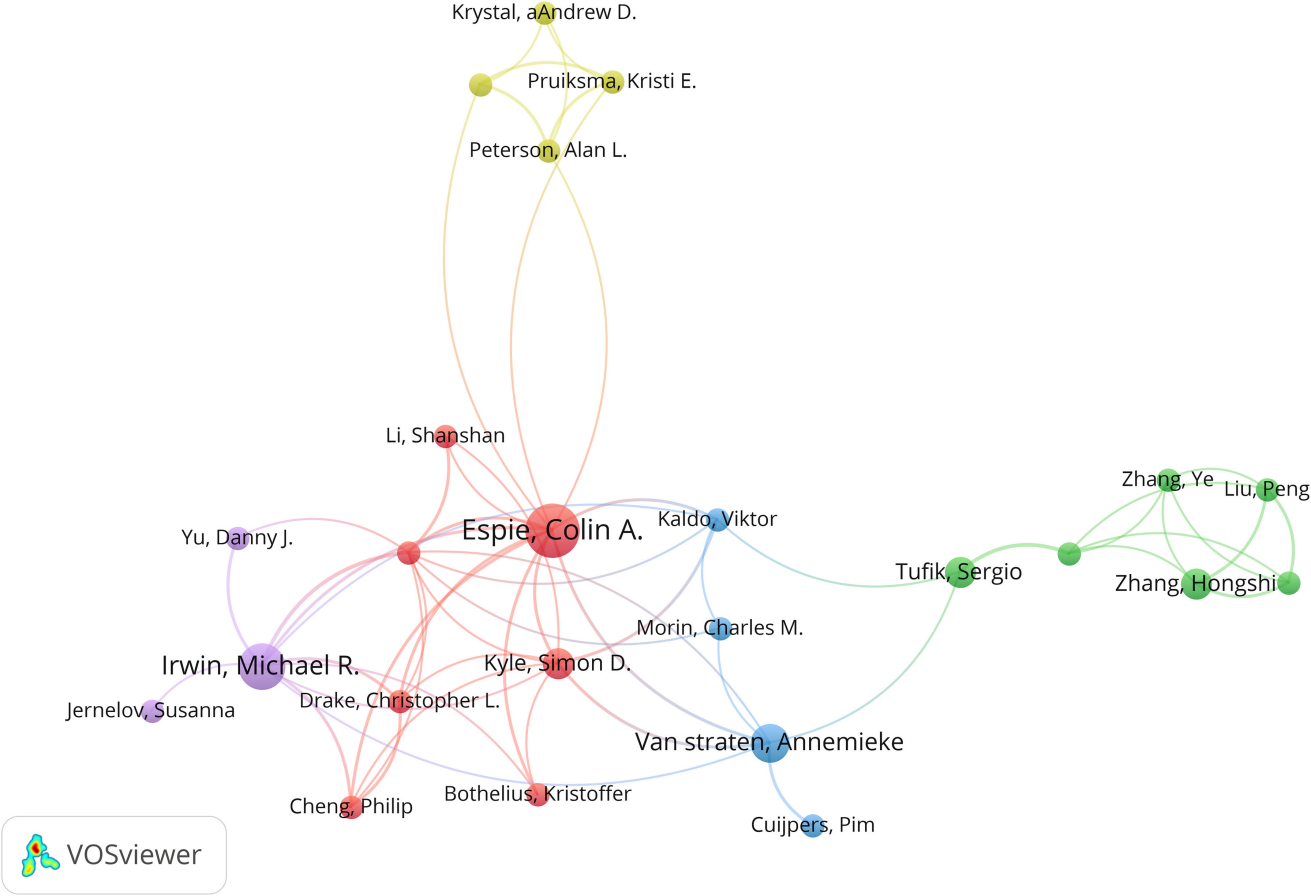
Co-cited authors network map.

To evaluate academic connections, 24 authors meeting the minimum threshold of 3 publications were analyzed. The resulting collaboration network (**Figure 7**) consists of 5 clusters. While a relatively stable core group has emerged (notably the red cluster comprising 7 authors), the overall collaborative network remains somewhat fragmented, indicating substantial room for enhancing cross-institutional collaboration.

### 3.4 Number of citations analysis

As a core indicator for measuring academic value, the citation frequency of literature typically reflects its greater influence in the respective research field when the frequency is higher. **Table 5** summarizes the top 10 highly influential literature items with the highest citation frequencies. Among them, the literature with the highest citation frequency and an impact factor of 6 is Clinical Practice Guidelines for the Prevention and Management of Pain, Agitation/Sedation, Delirium, Immobility, and Sleep Disruption in Adult Patients in the ICU, and its impact factor (IF) is 6 (23). The present work revises and augments the 2013 guidelines addressing pain, agitation and delirium in adult ICU populations. It explicitly demonstrates potential interrelationships between pain, agitation/sedation status, delirium, immobility and impaired sleep in critically ill adults. Beyond introducing innovative academic viewpoints, this study also deepens the recognition of sleep’s therapeutic and preventive effects on symptoms prevalent in critically ill patients. It is noteworthy that several highly cited references focus on general alternative medicine (e.g., herbal medicine) and first-line treatments like Cognitive Behavioral Therapy for Insomnia (CBT-I). This indicates that the intellectual base of manual therapy is deeply rooted in the broader context of complementary therapies and standard psychological interventions, serving as comparative benchmarks for evaluating manual therapy’s efficacy.

**Table 5:** Top 10 most cited search documents.

Title	First author	Source title	Total citations	Average per year	Impact factor (IF)(2024)
Clinical Practice Guidelines for the Prevention and Management of Pain, Agitation/Sedation, Delirium, Immobility, and Sleep Disruption in Adult Patients in the ICU	Devlin John W.	CRITICAL CARE MEDICINE	1145	143.13	6
Cognitive Behavioral Therapy for Chronic Insomnia A Systematic Review and Meta-analysis	Trauer James M.	ANNALS OF INTERNAL MEDICINE	699	63.55	15.4
Clinical Practice Guidelines on the Evidence-Based Use of Integrative Therapies During and After Breast Cancer Treatment	Greenlee Heather	CA-A CANCER JOURNAL FOR CLINICIANS	535	59.44	232.4
Clinical Guidelines for the Manual Titration of Positive Airway Pressure in Patients with Obstructive Sleep Apnea	Kushida Clete A.	JOURNAL OF CLINICAL SLEEP MEDICINE	486	27	2.9
Chamomile: A herbal medicine of the past with a bright future (Review)	Srivastava Janmejai K.	MOLECULAR MEDICINE REPORTS	428	26.75	3.5
The neurobiology, investigation, and treatment of chronic insomnia	Riemann Dieter	LANCET NEUROLOGY	414	37.64	45.5
Maternal-Preterm Skin-to-Skin Contact Enhances Child Physiologic Organization and Cognitive Control Across the First 10 Years of Life	Feldman Ruth	BIOLOGICAL PSYCHIATRY	396	33	9
Cognitive Behavioral Therapy for Insomnia Comorbid With Psychiatric and Medical Conditions A Meta-analysis	Wu Jade Q.	JAMA INTERNAL MEDICINE	356	32.36	23.3
A key role for orexin in panic anxiety	Johnson Philip L.	NATURE MEDICINE	313	19.56	50
Integrative Therapies During and After Breast Cancer Treatment: ASCO Endorsement of the SIO Clinical Practice Guideline	Lyman Gary H.	JOURNAL OF CLINICAL ONCOLOGY	281	35.13	43.4

### 3.5 Journals and co-cited journals

**Figure 8:**
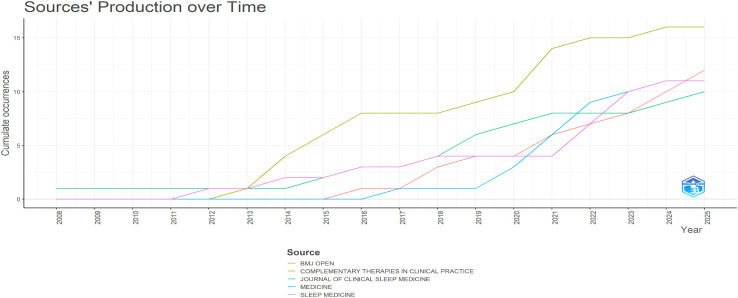
Trends in sources’ production over time.

**Figure 9:**
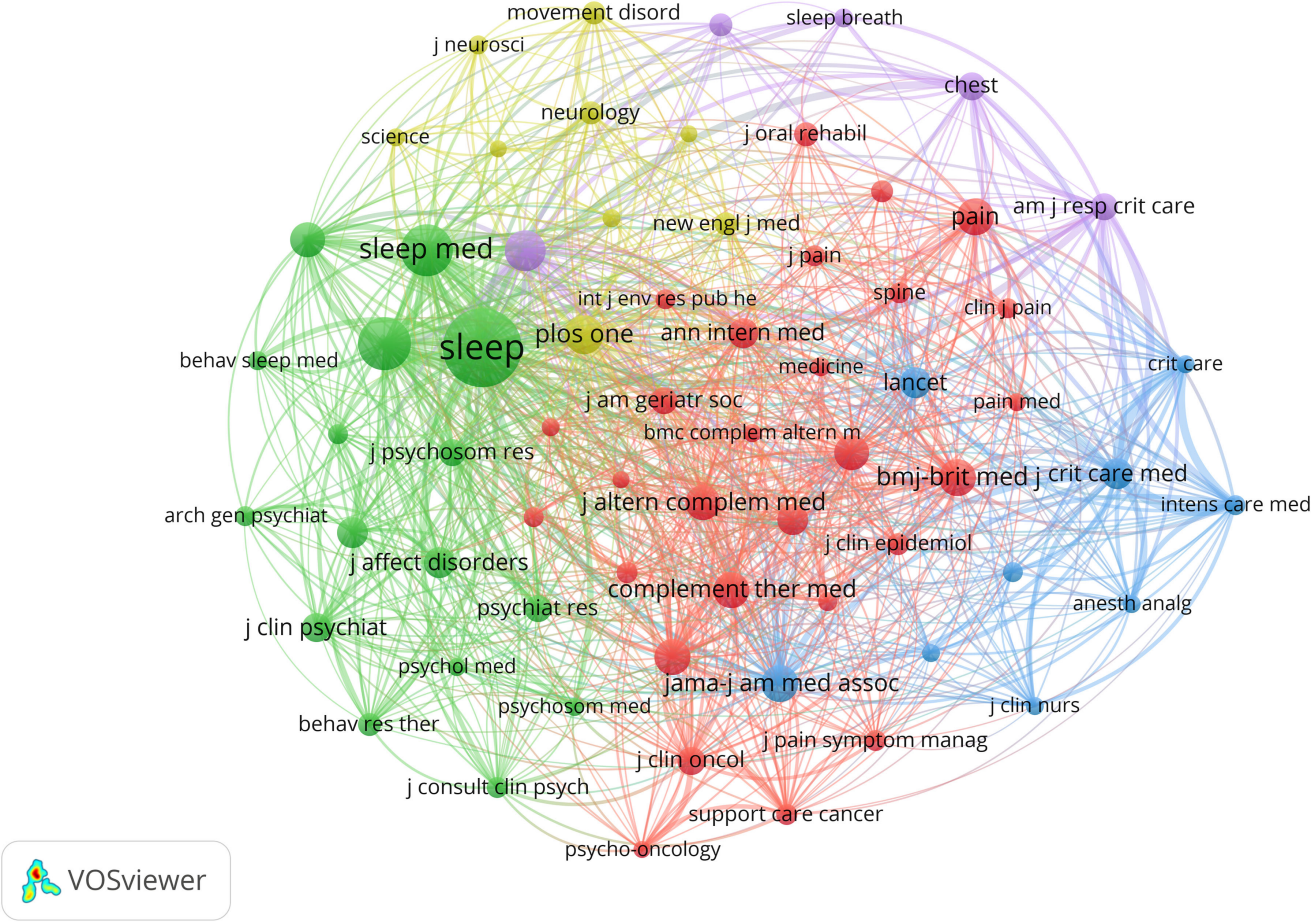
Web visualization for journal citation analysis.

This study reveals that a total of 594 academic articles related to manual therapy for sleep disorders have been published in 321 journals worldwide. **Figure 8** illustrates the longitudinal publication trends of the top five most prolific journals in this domain. All five journals exhibit a robust upward trajectory. Notably, Complementary Therapies in Clinical Practice not only ranks first in total publication volume but also displays the sharpest growth rate. The remaining four journals demonstrate a “stepwise growth” pattern: a steady climb from 2015 to 2020, followed by a markedly accelerated growth phase post-2020. This collective upward trend reflects the continuously enhancing academic influence and dissemination capacity of these journals regarding non-pharmacological sleep interventions. **Figure 9** displays the results of a journal co-citation analysis conducted using VOSviewer software. This analysis was restricted to journals with no fewer than 75 articles on the topic, ultimately selecting 67 eligible journals for inclusion. Ranked by Total Link Strength (TLS), the top five journals are *Sleep* (TLS = 30113), Critical Care Medicine (TLS = 16105), Sleep Medicine Reviews (TLS = 16045), Sleep Medicine (TLS = 15203), and Journal of Clinical Sleep Medicine (TLS = 10386). The journal that boasts the highest total link strength is Sleep, suggesting that it may be a leading or authoritative journal in the field.

### 3.6 Keywords and the evolution of keywords analysis

As a condensed extraction of the core content of literature, keyword analysis provides a crucial basis for exploring research hotspots and frontier directions in a field. **Table 6** summarizes the top 10 most frequently occurring keywords in this domain, among which “insomnia” and “sleep” rank prominently with high frequencies of 131 and 109 occurrences, respectively.

**Table 6:** Top 10 keywords ranked by frequency of occurrence in WoSCC.

Rank	Keywords	Occurrences
1	insomnia	131
2	sleep	109
3	depression	87
4	therapy	74
5	pain	73
6	anxiety	72
7	sleep quality	68
8	massage	67
9	management	58
10	symptoms	54

**Figure 10:**
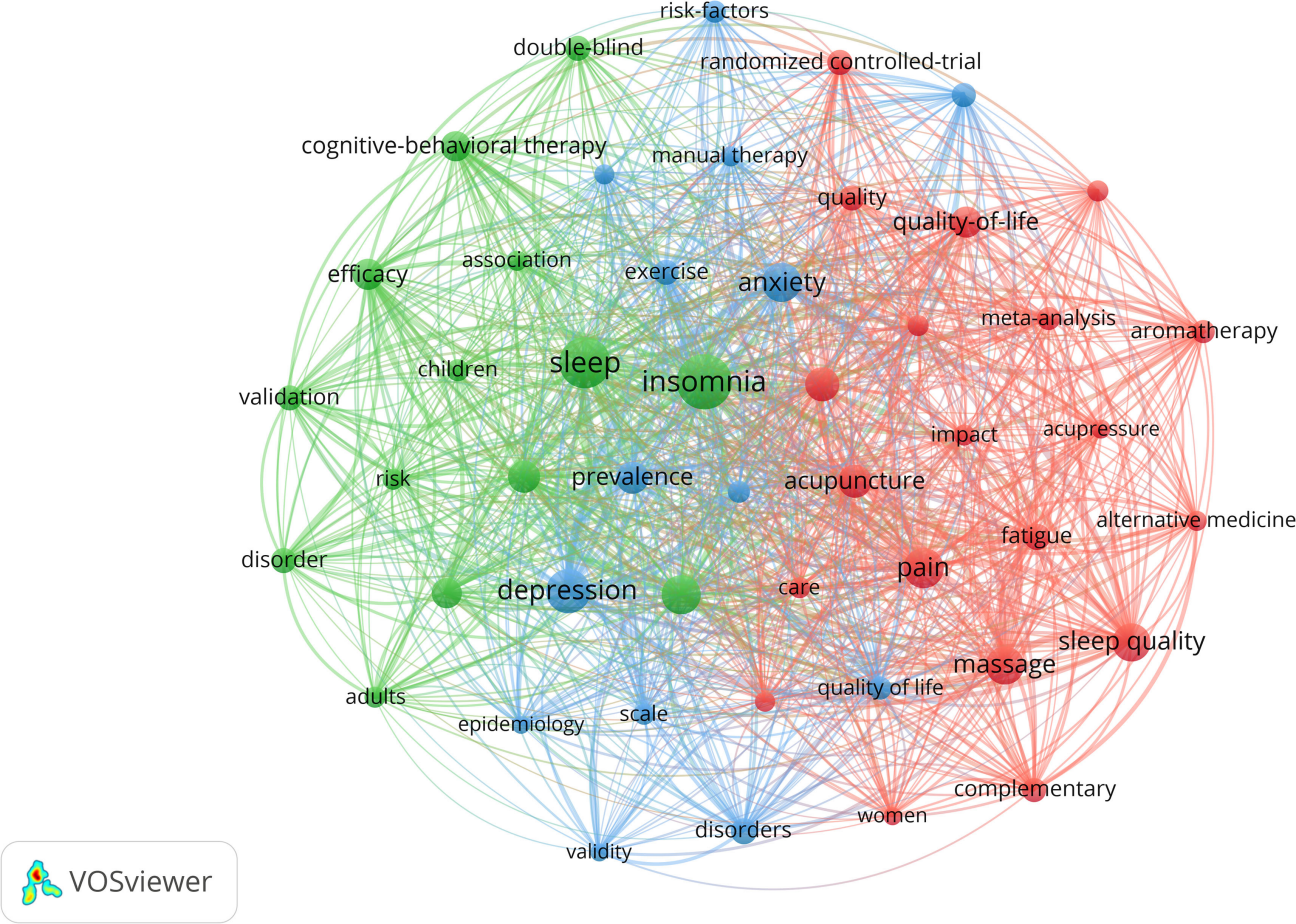
Keywords co-occurrence clustering map.

To further identify key nodes and research frontiers in the field, keyword co-occurrence analysis was conducted using VOSviewer software. A total of 3,333 keywords were retrieved by the software. Of these, 48 keywords with more than 20 occurrences were selected for clustering analysis. As shown in **Figure 10**, four clusters with distinct colors were finally formed. The red cluster is the largest, which includes 20 keywords such as “acupressure”, “massage”, “sleep quality”, “alternative medicine”, and “fatigue”. The green and blue clusters are equal in size, each containing 14 keywords. The green cluster focuses on themes like “sleep”, “insomnia”, “risk”, “association”, and “disorder”, while the blue cluster centers on content such as “anxiety”, “depression”, “manual therapy”, “prevalence”, and “quality of life.”

Combining keyword frequency and clustering characteristics, it is evident that current research in this field focuses on exploring the relationships between “sleep”, “insomnia”, “disorder” and “anxiety”, “massage”.

**Figure 11:**
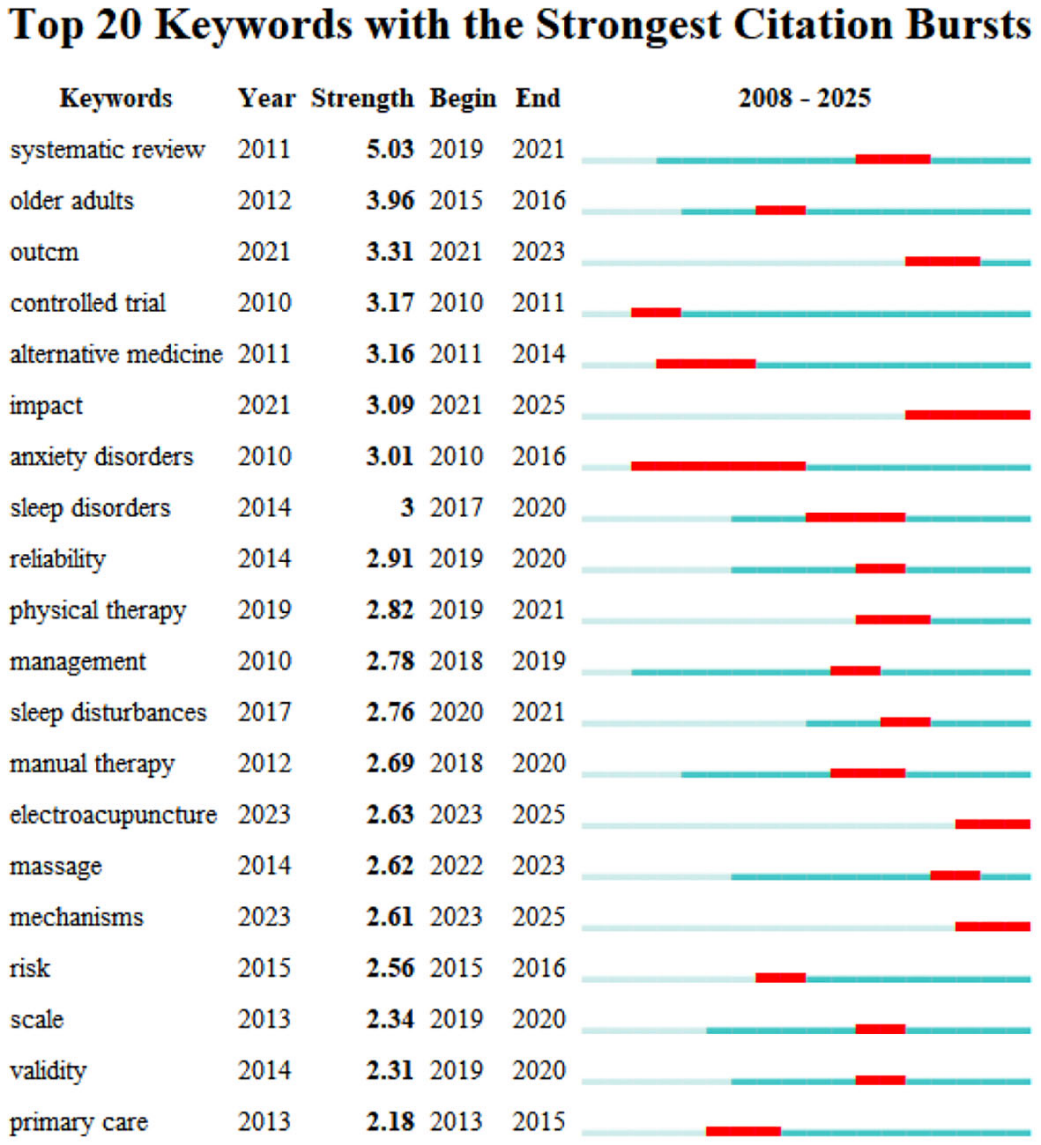
Top 20 keywords with the strongest citation bursts.

Keyword citation bursts can effectively reflect the popular fields and emerging themes of a discipline in specific periods. As shown in **Figure 11**, the analysis of keywords in the literature from 2008 to 2025 was conducted by CiteSpace software. The top 20 keywords with the strongest citation bursts were identified, and their citation burst characteristics during the research period were presented. Among them, “systematic review” and “older adults” exhibit the most significant burst strength. “Systematic review” started in 2011, with a citation burst occurring between 2019 and 2021. It reaches a strength of 5.03, ranking first. “Older adults” began in 2012, with a burst appearing in 2015–2016 and a strength of 3.96. The earliest keywords to show citation bursts are “controlled trial”, “anxiety disorders”, and “management”. Notably, “electroacupuncture” and “mechanisms” have maintained citation bursts from 2023 to 2025. As newly emerging keywords in recent years, they confirm that research in this field has gradually shifted from early clinical validation to the development of precision intervention techniques (such as electroacupuncture) and a deeper exploration of underlying mechanisms.

### 3.7 Co-cited references analysis

Co-cited references analysis involves analyzing related documents which are simultaneously cited by the same subsequent literature. Such documents typically share core academic characteristics or research attributes within the citation network. Specifically, through co-citation burst analysis, the core focus and development trends of specific research topics in different periods can be revealed.

**Figure 12:**
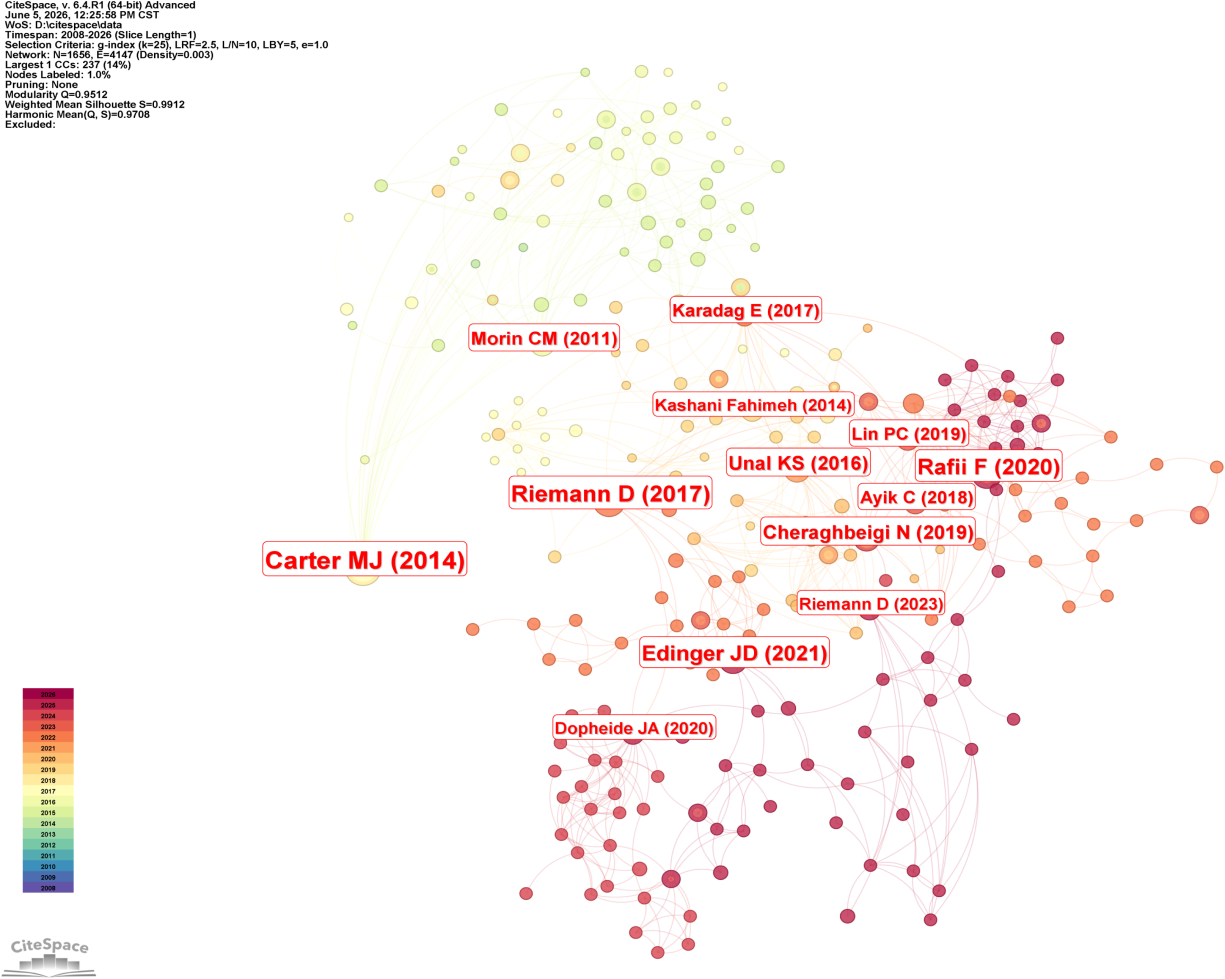
Co-cited references.

This study used CiteSpace software to analyze the co-citation of references in the field. The constructed visual co-citation network includes 1656 nodes (representing literature) and 4147 links (representing co-citation relationships). As shown in **Figure 12**, the dense areas from purple to red in the network are core clusters, such as the regions where literature like “Reaven D (2017)” and “Eltinger JD (2021)” are located. This intuitively reflects the close connections between these literatures in the field. These constitute the research hotspot literature or fundamental core literature of the domain.

**Figure 13:**
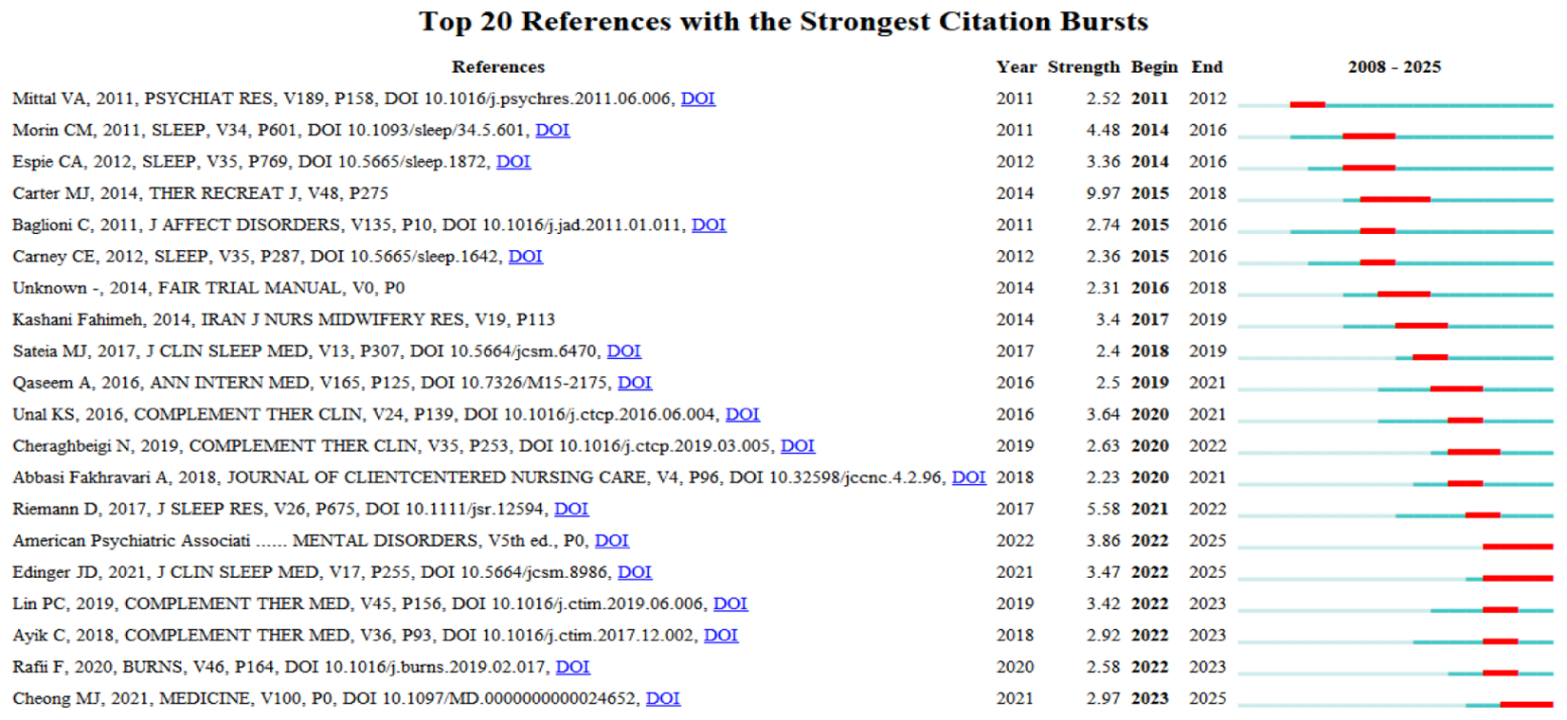
Top 20 references with the strongest citation bursts.

Analysis of references with strong citation bursts (**Figure 13**) reveals a predominant focus on core disciplines, including psychiatry, sleep medicine, and psychotherapy. These highly cited foundational papers have critically propelled the development of the disciplinary knowledge framework. By tracking the evolutionary trajectories of these citation bursts, we can gain clear insights into the dynamic shifting of research hotspots in manual therapy for sleep disorders.

Additional analysis demonstrates that these citation-bursting publications predominantly focus on core fields such as psychiatry, sleep medicine, and psychotherapy. As key literature frequently cited in the field, they have effectively promoted the development of the disciplinary knowledge system. By tracking the citation burst trajectories of this literature, we can gain insight into the dynamic evolution process of research hotspots in the field of manual therapy for sleep disorders.

### 3.8 Translational clinical assessment of manual therapy

To validate how macroscopic bibliometric trends translate into real-world clinical applications, a search of PubMed identified 41 relevant clinical trials. Due to limitations related to database sources and differences in literature types, the analysis of these clinical trial data is provided solely as supplementary evidence. Regarding trends in publication volume, the clinical trial data from PubMed align with the overall trend observed in the WoSCC database, both showing a steady annual increase. Most of the aforementioned 41 clinical trials were published after 2013 (**Supplementary Figure 2**). This timeline closely aligns with the pattern of a sharp increase in publications in the WoSCC database (**Figure 2**), confirming that manual therapy is gradually transitioning from basic theoretical research to the stage of clinical evidence. The high-frequency keywords listed in **Table 7** highlight the research characteristics of clinical trials, which are centered on participants and grounded in clinical efficacy. Among the 41 clinical trials, traditional Tuina was the primary intervention. Simultaneously, the keyword, such as massage and methods, indicates that various standardized and modified Tuina techniques are gradually being adopted. Regarding efficacy evaluation, in addition to subjective sleep scales such as the Pittsburgh Sleep Quality Index (PSQI), an increasing number of studies have introduced objective neuroendocrine indicators, such as cortisol, for assessment. This clinical profile exhibits substantial concordance with the bibliometric findings derived from the WoSCC database analysis presented in Section 3.6. To further elucidate the mechanistic rationale underlying tuina intervention for sleep disorders, this study subsequently investigated its putative mechanisms of action.

**Table 7:** Top 10 keywords ranked by frequency of occurrence in PubMed.

Rank	Keyword	Occurrences
1	humans	41
2	female	36
3	male	30
4	middle aged	29
5	adult	27
6	treatment outcome	20
7	aged	19
8	massage	16
9	massage/methods	9
10	quality of life	9

## 4 Discussion

### 4.1 Overview of main findings

This study systematically delineates the global research landscape and developmental trajectory of manual therapy for sleep disorders. From the perspective of national contributions, the United States emerged as the core contributing country with a 25.59% share of articles, followed by China (18.18%) and the United Kingdom (9.09%). These three countries not only dominate in terms of research output but also maintain close collaboration in academic cooperation. Co-authorship network analysis further confirms that the collaboration between China and the United States is particularly close. Through extensive academic collaboration, the two countries have jointly promoted the in-depth development of research in this field.

In the landscape of institutional research, Harvard University ranks first in institutional output with 12 published articles. However, compared to its high output, its collaborative links with other institutions are relatively weak. In contrast, Boston University plays a key role in cross-institutional collaboration, having led the formation of a tightly cooperative research cluster together with 8 other institutions, providing important support for the construction of collaborative networks within the field.

In terms of journal carriers, Complementary Therapies in Clinical Practice has become a core platform for publishing academic achievements in this field, with 16 relevant articles published. At the author level, Espie Colin A. is a highly influential core scholar in the field, leading with 6 publications and a total of 443 citations. The The European Insomnia Guideline: An update on the diagnosis and treatment of insomnia 2023 (24), which he co-authored, is a representative highly cited work. This guideline clearly states that antihistaminergic drugs, antipsychotics, immediate-release melatonin, ramelteon, and phytotherapies are not recommended for insomnia treatment in certain clinical scenarios, while it advocates for non-pharmacological treatments as auxiliary intervention methods for insomnia. This conclusion provides an important theoretical basis for the application of non-pharmacological approaches (such as manual therapy) in sleep-related diseases.

Regarding literature influence, the most frequently cited work is Clinical Practice Guidelines for the Prevention and Management of Pain, Agitation/Sedation, Delirium, Immobility, and Sleep Disruption in Adult Patients in the ICU (23), with a total of 1,145 citations. This guideline aims to update and expand the 2013 version of Clinical Practice Guidelines for the Management of Pain, Agitation, and Delirium in Adult Patients in the ICU. It clearly points out that “acupressure can improve sleep problems in critically ill patients to a certain extent”, providing key practical references for the application of manual therapy in the intervention of sleep disorders in special populations (critically ill patients).

Furthermore, keyword analysis further reveals the research focus of the field. The clustering characteristics of high-frequency keywords such as “insomnia”, “sleep”, and “depression” indicate that sleep-related diseases represented by insomnia have a strong correlation with depressive symptoms. The high-frequency occurrence of “massage” reflects that massage is widely recognized by the academic community for its effectiveness in the intervention of sleep-related diseases. It also confirms that the application value of manual therapy in this field is gradually becoming prominent.

### 4.2 Bibliometric insights into targeted sleep disorders

From a medical perspective, sleep disorders are not a single disease but a collective term for conditions characterized by “abnormal alterations in sleep patterns”. The disease is accompanied by overall health impairment (such as reduced daytime functioning and multi-system complications). Based on differences in pathogenesis and clinical manifestations, sleep disorders can be further subdivided into specific disease types including insomnia, narcolepsy, restless legs syndrome, and sleep apnea, providing a classification basis for precise diagnosis and targeted treatment (25).

The four most common sleep disorders encountered in clinical practice are insomnia, narcolepsy, restless legs syndrome (RLS), and sleep apnea. The keyword cluster analysis in the results section shows that “insomnia” is the most frequently occurring keyword. The fact that insomnia has become the most frequently searched keyword is determined by a combination of its epidemiological characteristics, clinical treatment needs, and the advantages of therapeutic interventions. Insomnia is the most common sleep disorder complaint in clinical settings. It exists either as an independent condition or co-occurring with other physical illnesses (such as chronic pain) or mental disorders (such as depression). Insomnia is accompanied by daytime impairments such as fatigue, low energy, mood disturbances, and cognitive complaints (26) and as the most prevalent type of sleep disorder globally, insomnia affects sleep health across different age groups and populations through its episodic, recurrent, or persistent course. It is clear that effective treatments for insomnia have become a hot topic and a growing trend in research. Cognitive Behavioral Therapy (CBT-I) for Insomnia disorder serves as the first-line non-pharmacological treatment, given its more durable effects and fewer adverse effects compared with pharmacological approaches (27). There is much empirical evidence to support the clinical efficacy of manual therapy for insomnia, especially cognitive behavioral therapy for insomnia (CBT-I), in managing insomnia in a wide range of populations (28). In summary, manual therapy for sleep disorders has become a major area of research.

### 4.3 The shift towards non-pharmacological and manual interventions

Bibliometric analysis of WoSCC revealed “treatment” as a high-frequency keyword in this field, indicating that the intervention and management of sleep disorders have emerged as a research priority of growing contemporary interest. The traditional treatment for sleep disorders is medication. In the field of pharmacotherapy, commonly used clinical interventions include benzodiazepines (BZ), benzodiazepine receptor agonists (BZRA), melatonin preparations, suvorexant, as well as sedative antidepressants and atypical antipsychotics (24). Pharmacotherapy offers distinct advantages, leveraging well-defined pharmacological mechanisms and rapid efficacy to provide symptomatic relief for various insomnia and related sleep disorders. However, pharmacological treatment faces significant clinical limitations. On one hand, achieving precise control over dosage and duration of use is challenging, requiring dynamic adjustments based on individual patient variations. On the other hand, excessive or prolonged use may lead to side effects such as drug dependence, daytime somnolence, and cognitive impairment, thereby limiting its long-term therapeutic value (24). Based on this, exploring safe and sustainable non-pharmacological treatment options has become an essential research direction in the field of sleep disorder treatment.

Compared to pharmacological treatments, non-pharmacological therapies offer the core advantages of high safety and low risk of side effects, employing general non-pharmacological approaches such as Cognitive Behavioral Therapy for insomnia (CBT-I), Physical therapy, manual therapy and traditional Chinese medicine (TCM) treatments (25).

CBT-I, backed by the most robust evidence-based medical support, is recommended as the first-line therapy for chronic insomnia disorder in adults of any age (29). However, its global promotion and clinical adoption face dual bottlenecks: high implementation costs and a shortage of specialized practitioners have resulted in low application coverage in primary healthcare settings and resource-limited regions (30).

Physical therapy encompasses a variety of treatment methods, including repetitive transcranial magnetic stimulation (rTMS), light therapy, and aromatherapy (25). The advantage of physical therapy lies in its ability to effectively treat sleep disorders while maintaining a high level of safety and a low incidence of adverse reactions. It can serve as an alternative to sedative and hypnotic medications, thereby reducing the risk of drug abuse or dependence on sedative and hypnotic treatments (31). However, at present, the application of physical therapy has not been widely adopted in clinical practice due to its unclear mechanisms (32).

Several supplementary interventions, including biofeedback, hypoglossal nerve stimulation, weighted blanket therapy, and gut microbiota-targeted interventions, have also been explored. While these approaches generally demonstrate favorable safety profiles, they are commonly constrained by limited adherence, elevated treatment costs, insufficient clinical evidence, or a lack of standardized administration protocols (15–17). Compared with the above treatments, manual therapy has certain strengths. It is a non-invasive physical intervention with almost no drug-related side effects or risk of addiction (33), and is a convenient operation with low economic burden, making it suitable for long-term rehabilitation and wide clinical promotion among patients with sleep disorders (34).

Manual therapy includes plenty of techniques such as chiropractic, shiatsu therapy and traditional Chinese tuina massage. Tuina massage is one of the most extensively researched and applied therapeutic techniques. Traditional Chinese treatments include not only massage but also acupuncture and Chinese-style exercises (35). Accumulated clinical evidence in recent years indicates that distinctive traditional Chinese medicine therapies are gaining increasing recognition and widespread application in treating sleep disorders. Particularly, traditional external therapies such as acupuncture and tuina massage—developed based on meridian and zang-fu organ theories—offer advantages including strong clinical efficacy and minimal adverse reactions.

By massaging specific acupoints, tuina regulates the body’s qi and blood circulation, thereby improving sleep quality and duration. Research indicates that tuina treatment significantly reduces scores on the Pittsburgh Sleep Quality Index (PSQI) and the Insomnia Severity Index (38). Tuina ameliorates insomnia through a dual neuro-endocrine mechanism: acupoint stimulation elevates central serotonin (5-HT) levels, modulates hyperactive hypothalamic-pituitary-adrenal (HPA) axis activity by downregulating corticotropin-releasing hormone (CRH) and adrenocorticotropic hormone (ACTH), and upregulates dopamine receptor expression. These findings provide biological evidence supporting the non-pharmacological application of tuina in insomnia management (38, 39).

### 4.4 Emerging frontiers: deciphering the neurobiological mechanisms

Sleep, as a complex physiological rhythmic process in the human body, is regulated by a multifaceted network of factors. This network encompasses both internal physiological mechanism, such as neuroendocrine regulation and neurotransmitter balance. The external variables including environmental influences (e.g., light exposure, noise) and behavioral patterns (e.g., sleep schedules, stress levels). Together, these elements form a multidimensional, dynamic regulatory system (40).

Currently, sleep health issues have become a global public health challenge. It affects ordinary individuals across different ages, occupations, and regions, with incidence rates showing an upward trend year by year. This situation has made “elucidating the pathogenesis of sleep disorders” and “innovating intervention and treatment approaches” core research hotspots and frontier directions in the public health field. Both academia and society have attracted widespread attention.

The keyword “mechanisms” exhibited citation bursts in 2023, rapidly emerging as a cutting-edge hotspot within the field. This shift indicates a progressive reorientation of research focus toward in-depth investigation of the underlying mechanisms of therapeutic interventions, with mechanistic elucidation having evolved into a central and sustained research trajectory in both current and future scholarship. In-depth keyword analysis reveals that the clustering characteristics of high-frequency keywords such as “insomnia”, “sleep”, “depression”, “massage”, and “pain” clearly point to current research on sleep disorders focuses. The frequent appearance of terms such as “depression” and “pain” reveals that sleep can be disrupted by many medical or psychiatric conditions and insomnia disorder is commonly comorbid with chronic health conditions that themselves also affect sleep, such as anxiety, depression, and pain (41). This explains why insomnia becomes common in our daily life, compelling people to take it seriously and prompting extensive research into how to manage sleep disorders.

Internationally, there is considerable debate regarding the effectiveness of different therapeutic approaches for treating sleep disorders. The United States, which ranks among the top in terms of research publications, advocates that CBT-I should be the first-line treatment for insomnia (42). China, as the second-highest contributor of research publication, is committed to studying traditional Chinese massage as an alternative manual therapy for treating sleep disorders. Relevant research data indicates that traditional Chinese massage demonstrates certain efficacy in treating sleep disorders (35). Nevertheless, the clinical research on manual therapy is still in the early phase. Current relevant studies have multiple shortcomings, and high-grade evidence is far from adequate when compared with CBT-I. Further rigorous clinical trials are therefore required to strengthen its evidence base.

**Figure 14:**
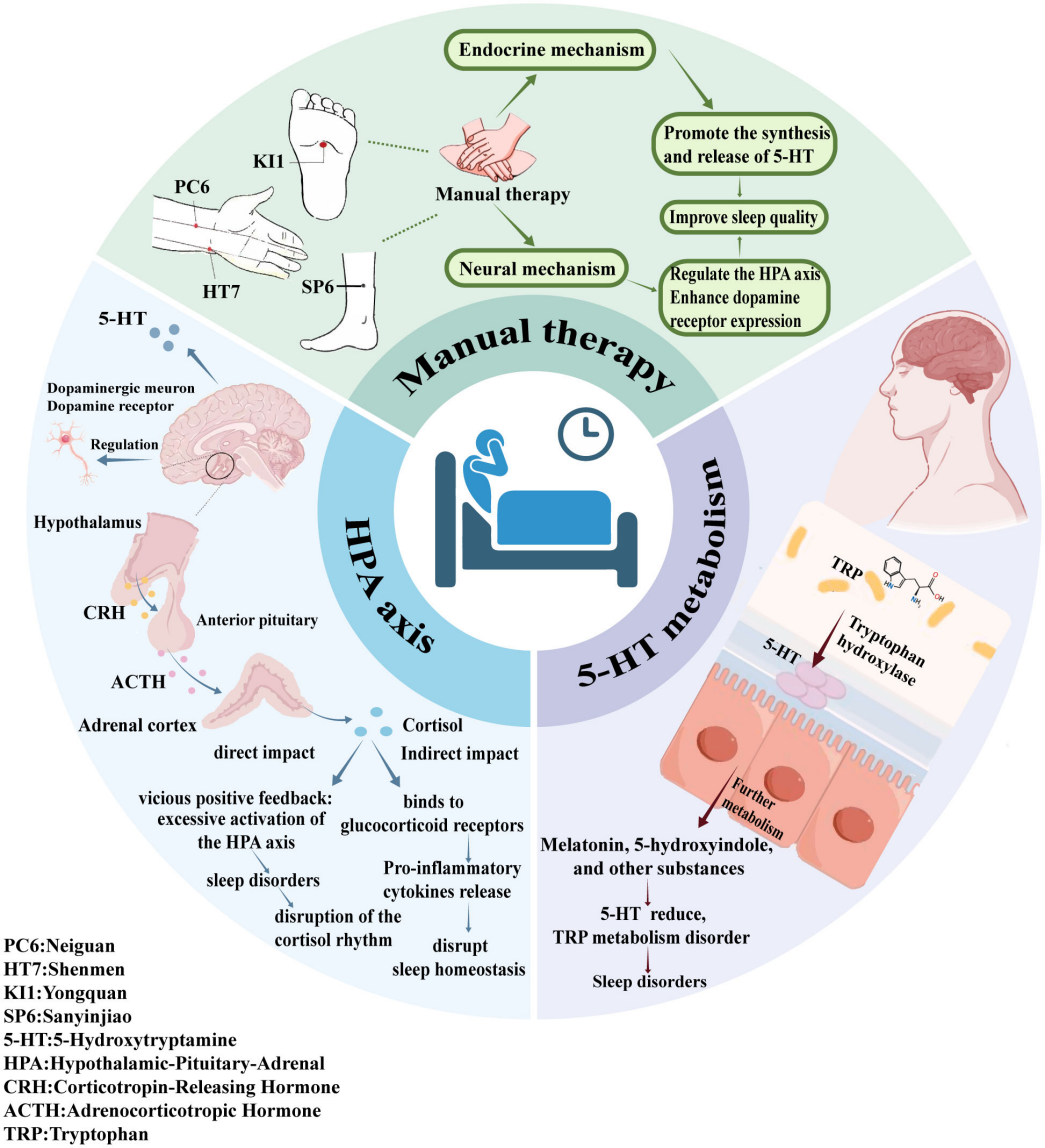
The pathogenesis of sleep disorders.

As reflected in bibliometric results, tuina therapy stands out among Traditional Chinese Medicine therapies and has become a core research focus. The high-frequency keywords “depression”, “anxiety” and “stress” identified in keyword co-occurrence analysis directly reveal that existing studies predominantly focus on emotional regulation. Emotions such as anxiety and depression accelerate the reuptake of 5-HT at the presynaptic membrane and the rate of peripheral 5-HT degradation. A comprehensive disruption of the entire 5-HT metabolic pathway leads to a deficiency of 5-HT in the central nervous system (36). At the same time, this disrupts the negative feedback of the HPA axis, resulting in a compensatory increase in cortisol levels and triggering persistent hyperactivity of the HPA axis (37). This indicates that emotion regulation is closely linked to 5-HT metabolism and HPA axis function. Tuina massage, as a non-pharmacological intervention, can improve sleep quality by regulating the aforementioned sleep-related mechanisms through multiple pathways and targets. Acupoint massage is a core intervention with its mechanism of action rooted in the central concept of Traditional Chinese Medicine—qi. Qi represents the vital energy sustaining living organisms, and sleep disorders arise from disharmonious internal qi flow. Acupoint massage aims to regulate qi flow within the body or specific organs, mobilizing the body’s natural healing capacity to resolve pathological conditions and restore health. Common acupoints for improving sleep quality include: Shenmen (HT7), Yongquan (KI1), Neiguan (PC6), and Sanyinjiao (SP6). Stimulating these points through pressure promotes functional changes in the peripheral and central nervous systems while altering cytokine and neurotransmitter circulation (34). **Figure 14** describes the pathogenesis of sleep disorders.

As an endocrine mechanism, massage therapy regulates HPA axis activity and suppresses its excessive activation through mechanical stimulation of skin and deep soft tissue receptors. It also reduces levels of key hormones such as corticotropin-releasing hormone (CRH), adrenocorticotropic hormone (ACTH), and cortisol (38). At the same time, it enhances the expression of dopamine D1 and D2 receptors as well as serotonin within the HPA axis, thereby improving sleep quality (43). The hypothalamic-pituitary-adrenal axis (HPA axis), serving as the core neuroendocrine pathway for the body’s acute stress response, exhibits a close bidirectional regulatory relationship with the onset and progression of sleep disorders. When the body encounters physiological stressors (e.g., trauma, infection) or psychological stressors (e.g., anxiety, pressure), the HPA axis is rapidly activated, initiating a cascade of hormonal regulatory responses: First, the paraventricular nucleus of the hypothalamus secretes corticotropin-releasing hormone (CRH). CRH acts on the anterior pituitary gland via the portal venous system, stimulating the release of adrenocorticotropic hormone (ACTH). Subsequently, ACTH is transported through the systemic circulation to the adrenal cortex, promoting the synthesis and release of cortisol. As the terminal effector hormone of the HPA axis, cortisol plays multiple critical roles in maintaining stress homeostasis, regulating metabolism, and modulating immune function. However, abnormal cortisol levels directly impact the sleep process (44). Specifically, excessive activation of the HPA axis during the early stages of sleep significantly increases sleep fragmentation, leading to reduced sleep continuity and subsequently triggering sleep deprivation. This sleep deprivation, in turn, promotes nocturnal cortisol secretion rhythm disruption, forming a vicious cycle of “HPA axis activation - sleep disturbance.” Furthermore, cortisol modulates the balance of cytokine networks by binding to glucocorticoid receptors on immune cell surfaces. This manifests as promoting the release of pro-inflammatory cytokines (such as tumor necrosis factor-α and interleukin-6) that disrupt sleep, further undermining sleep homeostasis. Animal studies have also confirmed that HPA axis dysfunction can indirectly interfere with central sleep regulatory networks by modulating dopaminergic neuron activity and affecting the expression levels of dopamine D1 and D2 receptors and 5-HT, ultimately leading to sleep disorders (45). Behavioral tests of the mice indicated that sleep desynchrony results from impaired HPA axis regulation (46). As can be seen, the HPA axis is closely linked to the regulation of sleep.

As a neural mechanism, this process promotes 5-HT synthesis and release, elevating central 5-HT levels, restoring neurotransmitter system balance, and ultimately improving sleep architecture and quality (38). Serotonin (5-hydroxytryptamine, 5-HT), a key monoamine neurotransmitter in the central nervous system, plays a central role in regulating higher neural activities such as the sleep-wake cycle, cognitive function, and emotional homeostasis. It occupies an irreplaceable position, particularly within sleep regulation pathways. Research has confirmed that activation of 5-HT neurons can induce arousal responses during non-rapid eye movement (NREM) sleep stages by regulating central sleep networks, thereby influencing sleep architecture. Metabolically, tryptophan—the precursor for 5-HT synthesis—is converted into 5-HT through the action of key enzymes such as tryptophan hydroxylase (44). Subsequently, 5-HT can undergo further metabolic pathways to generate downstream neuroactive substances such as melatonin and 5-hydroxyindole (47). When this metabolic process becomes abnormal, it leads to disrupted tryptophan metabolism accompanied by decreased 5-HT levels. Together, these factors can disrupt the neurochemical balance regulating central sleep control, ultimately inducing or exacerbating sleep disorders. Animal studies have shown that cats were rendered insomniac following the injection of a 5-HT synthesis inhibitor (48); 5-HT_2A_ and 5-HT_2C_ receptor knockout mice show a significant increase of wake and a reduction of slow wave sleep (SWS) (49). Both findings demonstrate that 5-HT plays a crucial role in the regulation of sleep.

### 4.5 Challenges in clinical translation

Relevant clinical trials have shown that traditional Chinese massage techniques, acupressure play a significant role in treating sleep disorders. Clinical data indicate that massaging multiple acupoints, such as Yongquan (KI1) and Shenmen (HT7), can effectively improve sleep (50). Additionally, relevant studies indicate that massaging the Baihui (DU20), Fengchi (GB20), and Anmian (EX-HN16) acupoints on the head, along with the Shenmen (HT7) acupoint on the hand, can effectively improve sleep. The significant improvement in the Pittsburgh Sleep Quality Index (PSQI) scores after testing serves as the best proof (51).

A review of the PubMed database reveals that the analysis of these studies highlights key trends and areas of focus. Current clinical trials primarily employ randomized controlled trials (RCTs), with assessments conducted through various methods including the PSQI and polysomnography (PSG). Among these, the PSQI serves as a classic clinical assessment tool for evaluating sleep quality in insomnia patients. This scale comprises 19 self-report items and 5 observer-rated items, with only the 18 self-report items contributing to the final score. The scoring items can be categorized into seven dimensions: sleep quality, sleep onset latency, sleep duration, sleep efficiency, sleep disturbances, hypnotic medication use, and daytime dysfunction. Each dimension is scored on a four-point scale (0–3 points: none, mild, moderate, severe). The sum of scores across all dimensions yields the PSQI total score (range 0–21 points). A higher total score indicates poorer sleep quality, providing a subjective assessment basis for insomnia diagnosis and severity grading (52). Additionally, PSG serves as the fundamental diagnostic tool for evaluating sleep-disordered breathing conditions. This noninvasive monitoring technique synchronously collects multiple physiological parameters throughout the entire night’s sleep, specifically including sleep stage classification, eye movements, electromyographic muscle tension, respiratory parameters, and electrocardiogram readings. By collecting these data, PSG enables quantification and assessment of the severity of sleep-related breathing disorders (SRBD). It also facilitates evaluation of abnormal sleep conditions such as central nervous system hypersomnia (including narcolepsy and idiopathic hypersomnia) and parasomnias (particularly rapid eye movement sleep behavior disorder) (53).

RCTs minimize various biases that may arise in the design and implementation of clinical trials, serving as the gold standard for evaluating the effectiveness of interventions (54). The implementation of blinding is crucial for ensuring the high quality of RCTs, while the appropriate design of placebo-controlled groups is a prerequisite for generating high-quality evidence from RCTs. Blind massage techniques are referred to as placebo massage techniques in clinical trials of massage therapy. The purpose is to simulate real massage therapy to form a control design and does not possess therapeutic effects. The internationally common comfort massage technique is the light touch method, wherein the practitioner lightly touches the patient’s skin without applying pressure to control the non-specific effects of the massage technique (55). However, this light touch shares similarities with certain techniques in massage therapy and may yield therapeutic effects. This makes it challenging to apply the blind method in clinical trials of massage therapy. This often results in a lack of reporting on key details of the placebo group in clinical studies, undermining the credibility of trial outcomes and hindering the advancement of massage therapy in clinical practice.

### 4.6 Advantages and limitations

This study conducts a bibliometric analysis on manual therapy for sleep disorders, with data extracted from the Web of Science Core Collection (WOSCC) database. While numerous literature databases are available for academic research, WOSCC was ultimately selected as the data source due to its three core advantages. First, it offers comprehensive and precise search capabilities, covering multidisciplinary and multi-type literature resources. The data inclusion authority and academic recognition lead globally. Second, its built-in search and analysis tools efficiently filter target literature aligned with the research theme, significantly enhancing data extraction accuracy and efficiency. Third, it supports fundamental visualization analysis, intuitively presenting key information such as publication trends within the field and institutional/author collaboration networks, laying the groundwork for subsequent in-depth analysis. Nevertheless, this study has limitations that must be considered when interpreting the results. First, data coverage remains incomplete. While the WOSCC database is extensive, its inherent inclusion criteria and scope limitations mean the extracted data may not fully encompass all global literature on manual therapy for sleep disorders. This potentially excludes high-quality research, potentially affecting the comprehensiveness of analytical findings. Second, bibliometric tools typically rely on author name abbreviations for matching, failing to distinguish between authors with identical abbreviations but different identities. This limitation may introduce biases in author publication volume statistics and collaboration analysis, potentially affecting the precision of research conclusions. Finally, research in manual therapy for sleep disorders is undergoing rapid development. New findings, techniques, and theoretical frameworks may emerge shortly after this study’s data collection and analysis were completed. Such dynamic academic advancements could alter the research priorities, cognitive directions, and developmental trends revealed by the current analysis, thereby limiting the timeliness of the study’s conclusions. In addition, this study limited its search and analysis to Chinese and English-language literature. This language restriction inevitably results in language bias and may fail to include studies from South Asia, Southeast Asia and other regions with distinctive traditional manual therapy systems. Accordingly, the present study cannot fully reflect the overall research status across the world.

## 5 Conclusion

This study systematically reviews and summarizes the overall research progress, core hotspots, and cutting-edge trends in manual therapy for sleep disorders. In recent years, the number of high-quality academic publications in this field has shown a sustained upward trend. This objective indicator fully confirms that research on manual therapy for sleep disorders is in a rapid growth phase of disciplinary development, with its academic value and clinical application potential increasingly attracting attention from the academic community. Regarding the pathological mechanisms of sleep disorders, the serotonergic 5-hydroxytryptamine (5-HT) neurotransmitter system and the hypothalamic-pituitary-adrenal (HPA) axis have emerged as central targets and research frontiers for elucidating the mechanisms underlying sleep homeostasis disruption. These findings provide crucial theoretical foundations and research directions for clarifying the mechanisms of action of manual therapy.

Integrating bibliometric findings with clinical trial characteristics, research on manual therapy for sleep disorders is undergoing a critical standardization transition. To facilitate its integration into evidence-based sleep medicine, future studies should advance along two dimensions. On one hand, future research informed by bibliometric findings should leverage multidisciplinary and precision medicine approaches to explore integrative protocols combining manual therapy with CBT-I, biofeedback, and neuromodulation, thereby addressing the limitations of monotherapy in the context of emotion-related pathogenesis, sleep heterogeneity, and multimorbidity. Concurrently, targeted investigation into emerging hotspots such as the precise neurobiological mechanisms underlying manual therapy is warranted to address the current deficiencies in both the breadth and depth of mechanistic understanding. On the other hand, refining trial methodology through double-blind, placebo-controlled designs to isolate placebo effects, alongside standardizing manual parameters and therapeutic protocols to enhance data consistency and consolidate foundations for meta-analysis.

## Data Availability

The original contributions presented in the study are included in the article/**Supplementary Material**. Further inquiries can be directed to the corresponding authors.
