# Supplementary figures and images for: Global trends and neurobiological frontiers of manual therapy in sleep disorders: integrating bibliometrics with clinical evidence
Source: Front Psychiatry. 2026 Jun 24;17:1863957. doi: 10.3389/fpsyt.2026.1863957 (PMC13341811; doi:10.3389/fpsyt.2026.1863957)

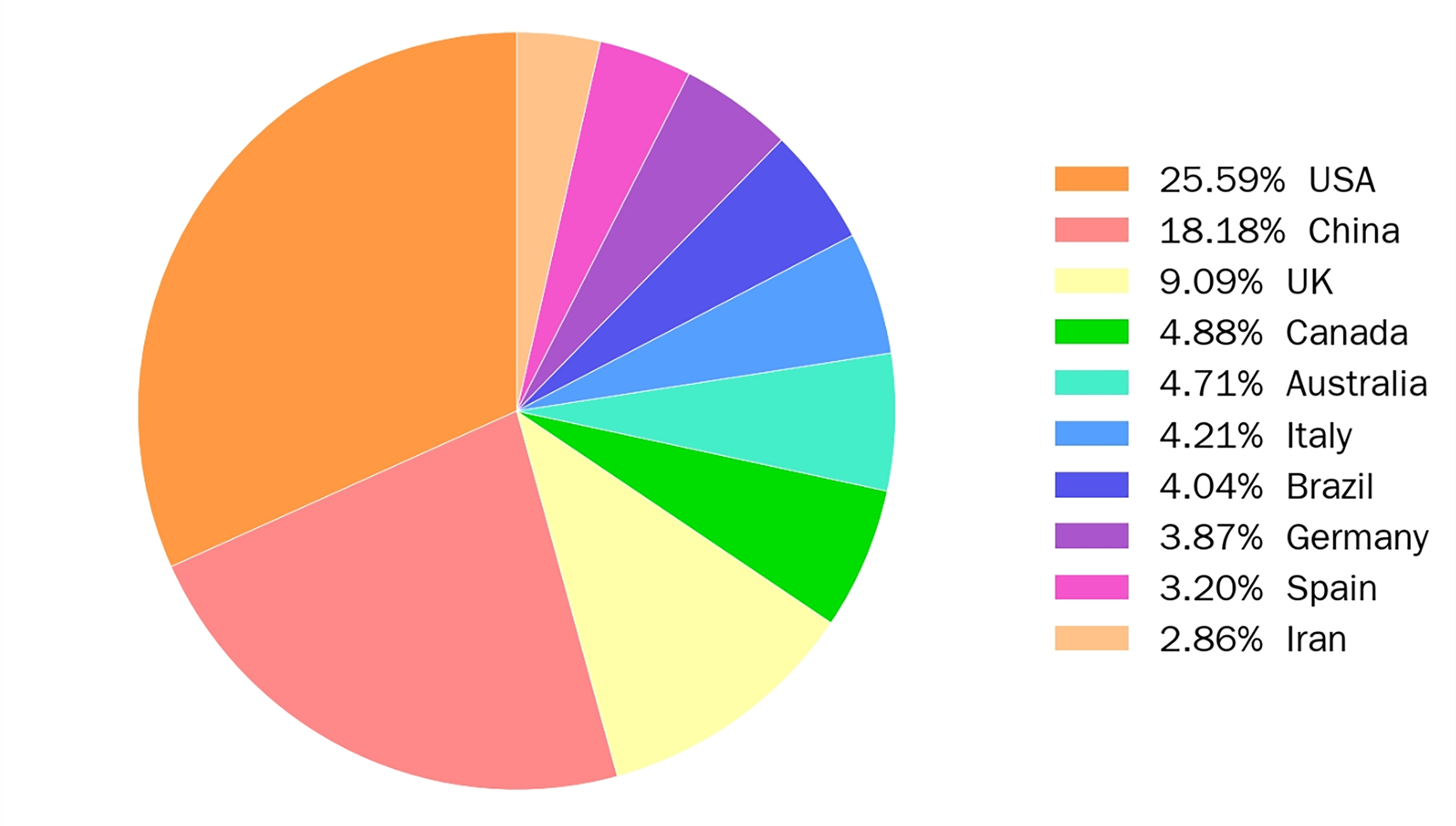

Supplement: Supplementary file 1 [file Image1.tif]

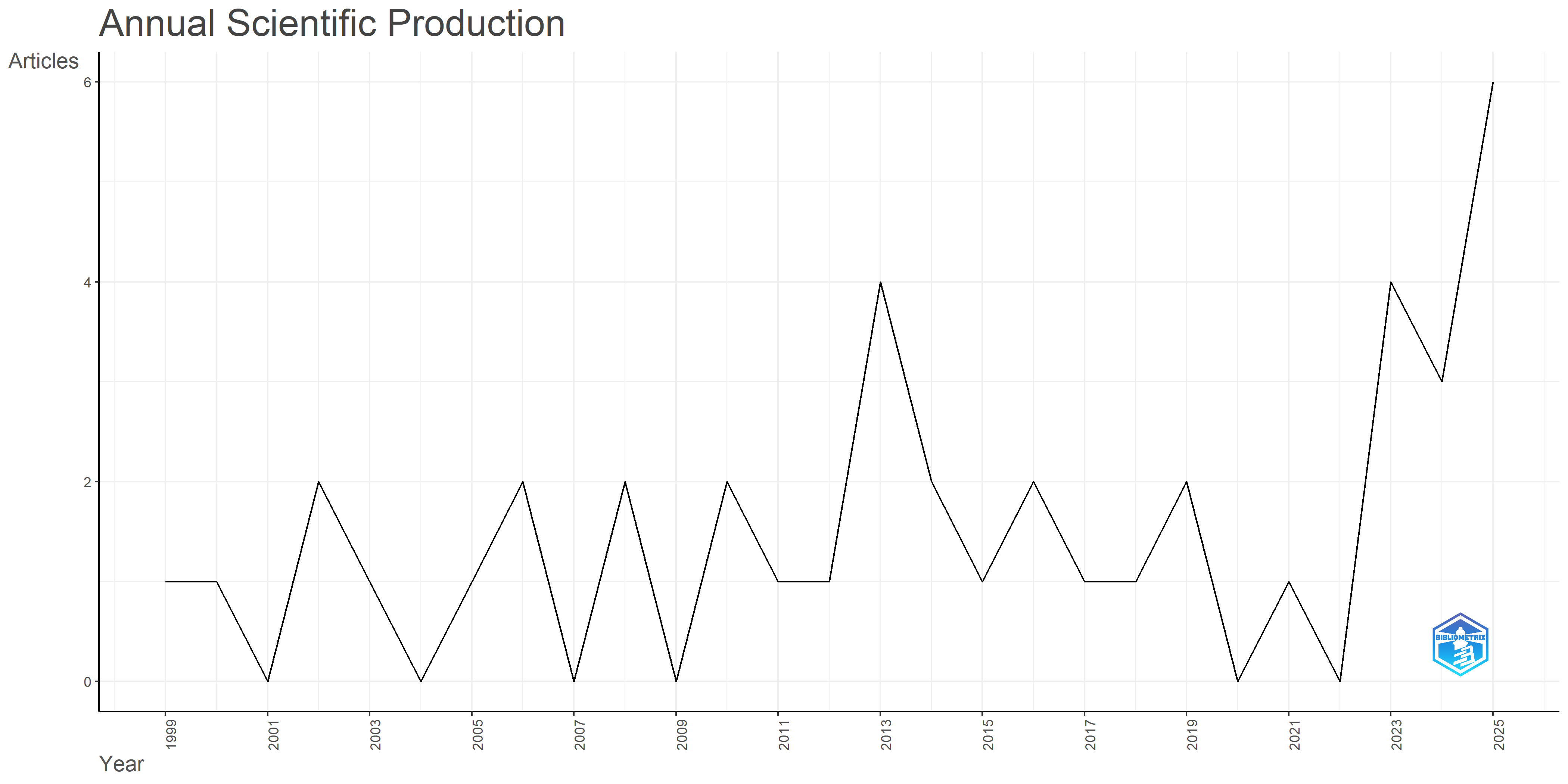

Supplement: Supplementary file 2 [file Image2.tif]
