# Supplementary material for: Global trends and neurobiological frontiers of manual therapy in sleep disorders: integrating bibliometrics with clinical evidence
Source: Front Psychiatry. 2026 Jun 24;17:1863957. doi: 10.3389/fpsyt.2026.1863957 (PMC13341811; doi:10.3389/fpsyt.2026.1863957)
Supplement: Supplementary file 3 [file SupplementaryFile1.docx]

**Identification of studies via databases and registers**

Records removed *before screening*:

Duplicate records removed (n = 0)

Records marked as ineligible by automation tools (n = 0)

Records removed for other reasons (n = 0)

Records identified from Web of Science Core Collection (WOSCC) AND PubMed

WOSCC (n = 608)

PubMed (n = 41)

**Identification**

Records screened

WOSCC (n = 608)

PubMed (n= 41)

Records excluded**

WOSCC (n = 14)

PubMed (n= 0)

Reports sought for retrieval

WOSCC (n = 594)

PubMed (n= 41)

Reports not retrieved

WOSCC (n = 0)

PubMed (n= 0)

**Screening**

Reports assessed for eligibility

WOSCC (n = 594)

PubMed (n= 41)

Reports excluded:

(n = 0)

Studies included in review

(n = WOSCC (n = 594)

PubMed (n= 41))

**Included**

*Consider, if feasible to do so, reporting the number of records identified from each database or register searched (rather than the total number across all databases/registers).

**If automation tools were used, indicate how many records were excluded by a human and how many were excluded by automation tools.

Source: Page MJ, et al. BMJ 2021;372:n71. doi: 10.1136/bmj.n71.

This work is licensed under CC BY 4.0. To view a copy of this license, visit <https://creativecommons.org/licenses/by/4.0/>
